# Supplementary material for: Social flocculation in plant–animal worms
Source: R Soc Open Sci. 2019 Mar 20;6(3):181626. doi: 10.1098/rsos.181626 (PMC6458428; doi:10.1098/rsos.181626)
Supplement: Supplementary Material [file rsos181626supp1.docx]

**Social Flocculation in Plant-Animal Worms**

Alan Worley, Ana B. Sendova-Franks & Nigel R. Franks

**Electronic Supplementary Material**

**List of supplemental sections:**

- **Section S1.** Aggregation during agitation
- **Section S2.** Bubble characteristics
- **Section S3.** Single worm speed estimation
- **Section S4.** Holes in the centres of toroidal flocs

**List of supplemental figures:**

- **Figure S1.** The extent of worm aggregation measured as the fraction of pixels due to worms, following the end of the agitation, using 15 fps video
- **Figure S2.** The extent of worm aggregation measured as the fraction of pixels due to worms, following the end of the agitation, using 2 fps time-lapse
- **Figure S3.** Experimental set-up for studying worm flocculation in a column of seawater
- **Figure S4.** The bubble at the start of its ascent during run #6
- **Figure S5.** The variation in the abundance of single worms passing through the water column measured as the number of pixels in a frame occupied by worms
- **Figure S6.** Aspect ratio h/L vs. L for the 22 flocs, where h and L are the mean minor and major axes, respectively, of the fitted ellipse
- **Figure S7.** Oblique views of toroidal flocs

**List of supplemental tables:**

- **Table S1.** Speed and dimensional data for 22 flocs of *S. roscoffensis* worms and the mean and standard deviation for the speed distributions of single worms in the same run

**List of supplementary videos:**

- **Video S1.** Video recording of flocculation of *Symsagittifera roscoffensis* worms in a culture flask filled with seawater

**Section S1. Aggregation during agitation**

We laid a polystyrene culture bottle (Corning 25 cm^2^ culture flask with a canted neck and a vent cap, product #430639) containing worms and filtered seawater on a white background and allowed the contents to settle. The bottle was then briefly shaken and replaced. The process was recorded by a fixed, overhead Canon G7 camera producing a video with a resolution of 768x1024 pixels at 15 fps (video S1). Individual images at 1s intervals were cropped to exclude all but the main part of the culture bottle, converted to binary and the fraction of pixels attributable to worms recorded as a measure of the extent of worm aggregation (figure S1). This fraction began at ~0.6; it was not possible to collect data during the shaking process, when it fell to ~0.38. When the agitation finished it rose to ~0.82 before slowly recovering to its starting value. The worms cluster together during agitation and rapidly disperse when it ends. We hypothesize that the worms are prepared to burrow into sand following disturbance, which they can only do as well-separated individuals. In the culture bottle, after the shaking and the separation of the worms is over, the pixel count falls as the worms revert to social groupings [1].

Similar results (figure 1 and figure S2) are obtained with time-lapse photography at 2 fps and greater resolution (2736 x 3648 pixels); for these runs, the images begin at the end of the agitation, so no value for the fraction of pixels due to worms before agitation is available.


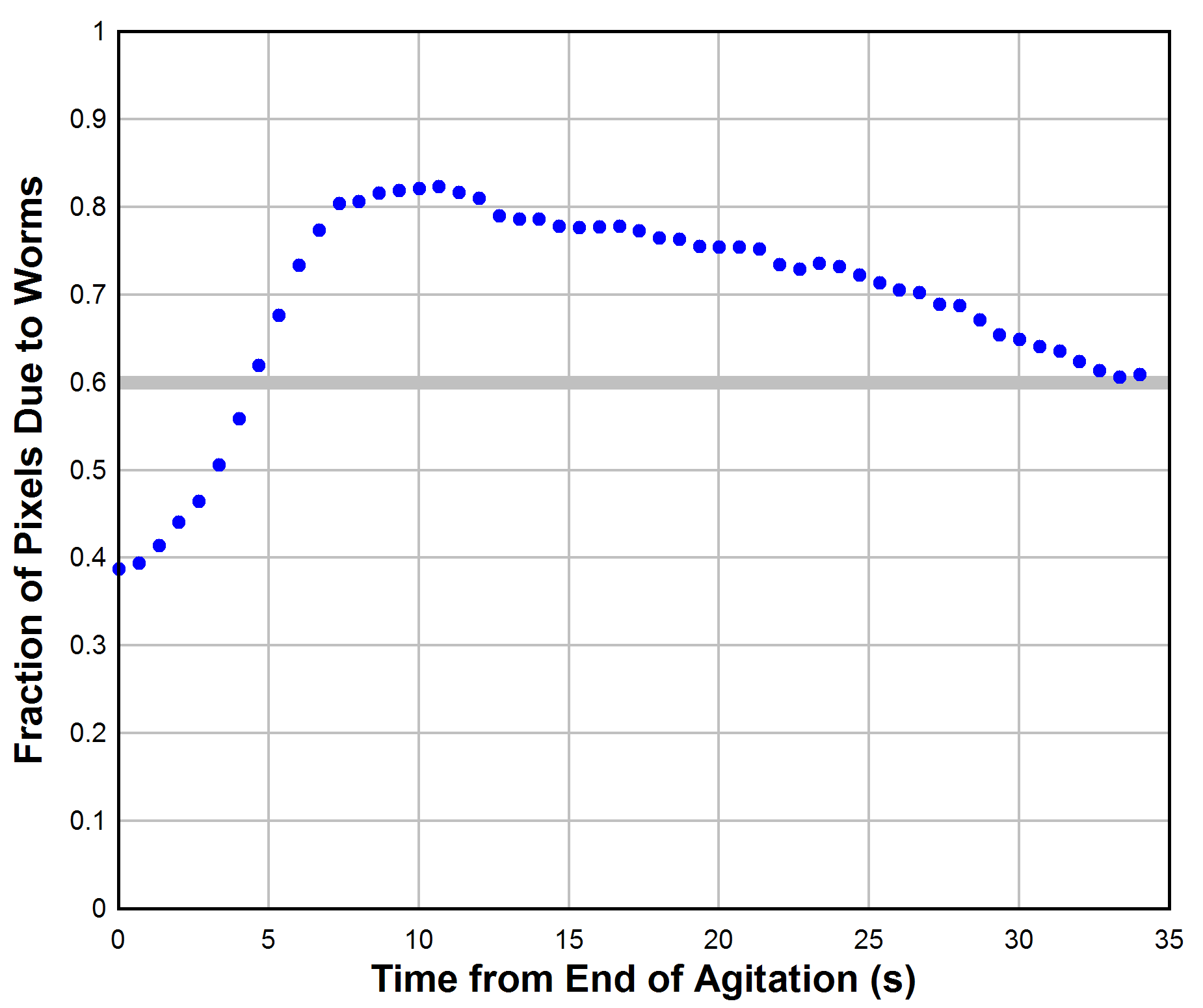


**Figure S1.** The extent of worm aggregation measured as the fraction of pixels due to worms, following the end of the agitation, using 15 fps video. The smaller the fraction of pixels due to worms, the greater is the extent of their aggregation. The grey line represents the initial and final values.


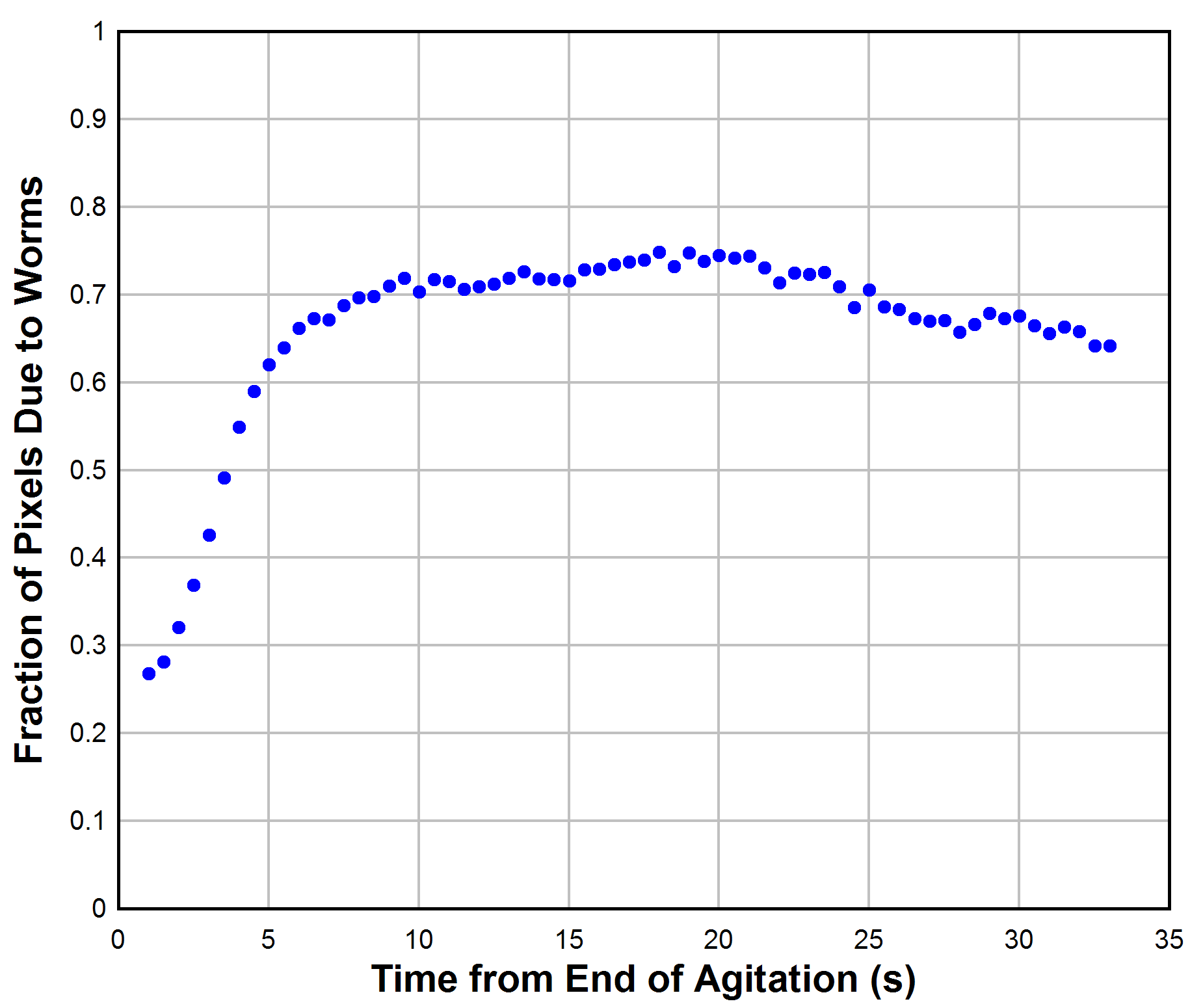


**Figure S2.** The extent of worm aggregation measured as the fraction of pixels due to worms, following the end of the agitation, using 2 fps time-lapse. The smaller the fraction of pixels due to worms, the greater is the extent of their aggregation.


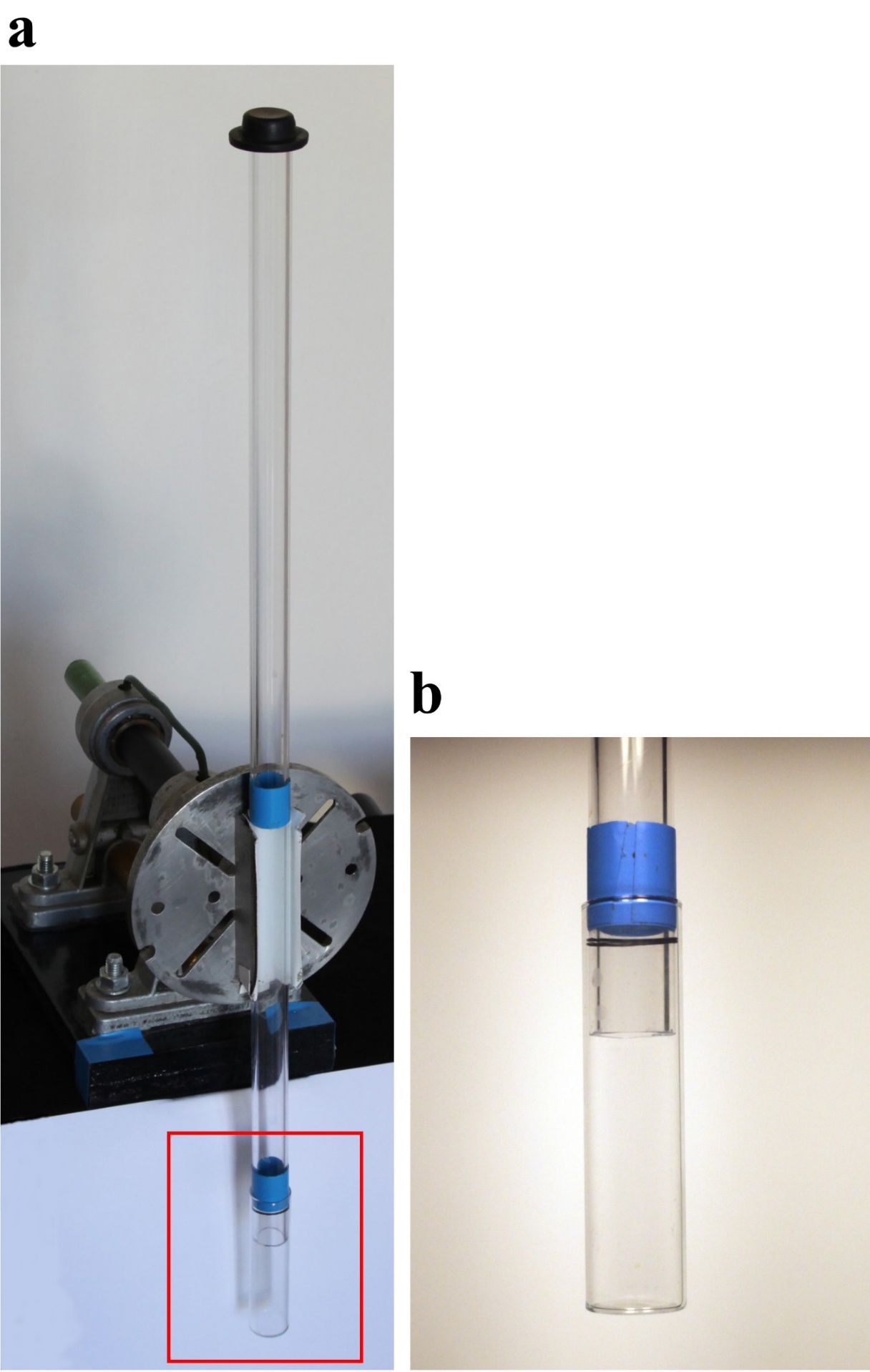


**Figure S3.** Experimental set-up for studying worm flocculation in a column of seawater. *(a)* Apparatus before rotation; the total column length is 555 mm. *(b)* Close-up view of the vial and O-ring seals.

**Section S2. Bubble characteristics**

The air bubble was found to play an important role. When the tube is inverted, the worms initially show only a low-level reaction. It is the arrival of the bubble which precipitates their activity and descent. For the floc measurements, video recording began before any movement of the apparatus was made. This allowed the rising bubble (figure S4) to be measured on its way to the top of the tube. The bubble height varied from 4.9 to 31.5 mm, 17.2 ± 8.0 mm (mean ± sd), n=16. One bubble was missed; this had no impact on the success of the run.

Independently of the floc runs, we investigated the transit time of the bubble, taking the initial time as the point when the tube reached the vertical after rotation, and the final time when the bubble reached the top. Two observers used stopwatches to time the transit for a range of bubble sizes. The two independent sets of timings were comparable and we have pooled them, 3.81 ± 0.32 s (mean ± s.d.), n = 33.


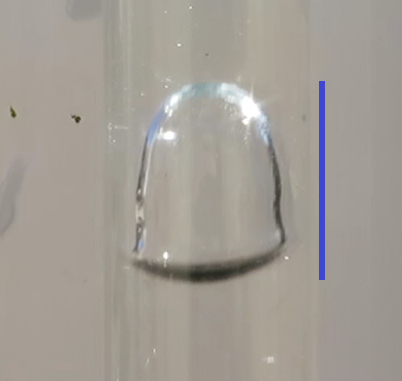


**Figure S4.** The bubble at the start of its ascent during run #6. The vertical line represents its measured height, 14.3 mm.

|  | | Flocs | | | | Single Worms | |
| --- | --- | --- | --- | --- | --- | --- | --- |
| Run # | Floc # | v (mm/s) | L (mm) | h (mm) | h/L | v (mm/s) | σ (mm/s) |
| 1 | 1 | 10.29 | 2.12 | 1.45 | 0.68 | 8.87 | 3.53 |
| 2 | 2 | 20.05 | 8.97 | 3.61 | 0.40 | 4.42 | 3.12 |
| 3 | 3 | 14.91 | 10.07 | 4.68 | 0.46 | 6.57 | 2.63 |
| 4 | 4 | 14.71 | 3.91 | 1.95 | 0.50 | 6.06 | 1.47 |
| 5 | 5 | 16.82 | 3.84 | 1.92 | 0.50 | 6.01 | 2.26 |
| 6 | 6 | 15.72 | 3.70 | 1.82 | 0.49 | 5.53 | 1.79 |
| 7 | 7 | 13.67 | 1.61 | 0.95 | 0.59 | 4.73 | 1.36 |
| 8 | 8 | 11.13 | 3.91 | 1.92 | 0.49 | 4.76 | 1.75 |
| 9 | 9 | 12.31 | 3.50 | 1.82 | 0.52 | 4.46 | 1.61 |
| 9 | 10 | 12.57 | 4.91 | 1.87 | 0.38 | 4.46 | 1.61 |
| 10 | 11 | 16.39 | 10.08 | 3.12 | 0.31 | 5.90 | 1.80 |
| 11 | 12 | 13.31 | 3.57 | 2.40 | 0.67 | 5.85 | 1.95 |
| 12 | 13 | 19.06 | 8.14 | 2.70 | 0.33 | 5.84 | 1.93 |
| 12 | 14 | 12.86 | 2.15 | 1.33 | 0.62 | 5.84 | 1.93 |
| 12 | 15 | 11.34 | 1.98 | 1.10 | 0.55 | 5.84 | 1.93 |
| 12 | 16 | 8.87 | 0.95 | 0.46 | 0.48 | 5.84 | 1.93 |
| 12 | 17 | 8.26 | 0.89 | 0.52 | 0.59 | 5.84 | 1.93 |
| 13 | 18 | 15.41 | 11.61 | 5.30 | 0.46 | 6.56 | 2.06 |
| 14 | 19 | 16.22 | 6.36 | 2.91 | 0.46 | 6.68 | 1.96 |
| 15 | 20 | 10.94 | 6.27 | 2.75 | 0.44 | 6.75 | 3.23 |
| 16 | 21 | 17.21 | 3.69 | 1.40 | 0.38 | 5.16 | 2.30 |
| 17 | 22 | 9.74 | 2.17 | 0.98 | 0.45 | 5.52 | 2.66 |

**Table S1.** Speed and dimensional data for 22 flocs of *S. roscoffensis* worms and the mean and standard deviation for the speed distributions of single worms in the same run. One run (#12) provides five flocs; L: mean major axis for the fitted ellipse; h: mean minor axis; v: velocity; σ: standard deviation.

**Section S3. Single worm speed estimation**

Figure S5 shows the result of fitting the single worm density for the same run as the one featuring floc #22, using a Gaussian speed distribution with mean 5.52 mm/s and standard deviation 2.66 mm/s. The measured terminal velocity of the floc itself is 9.74 mm/s. The results for the 17 separate runs containing 22 flocs are included in table S1.


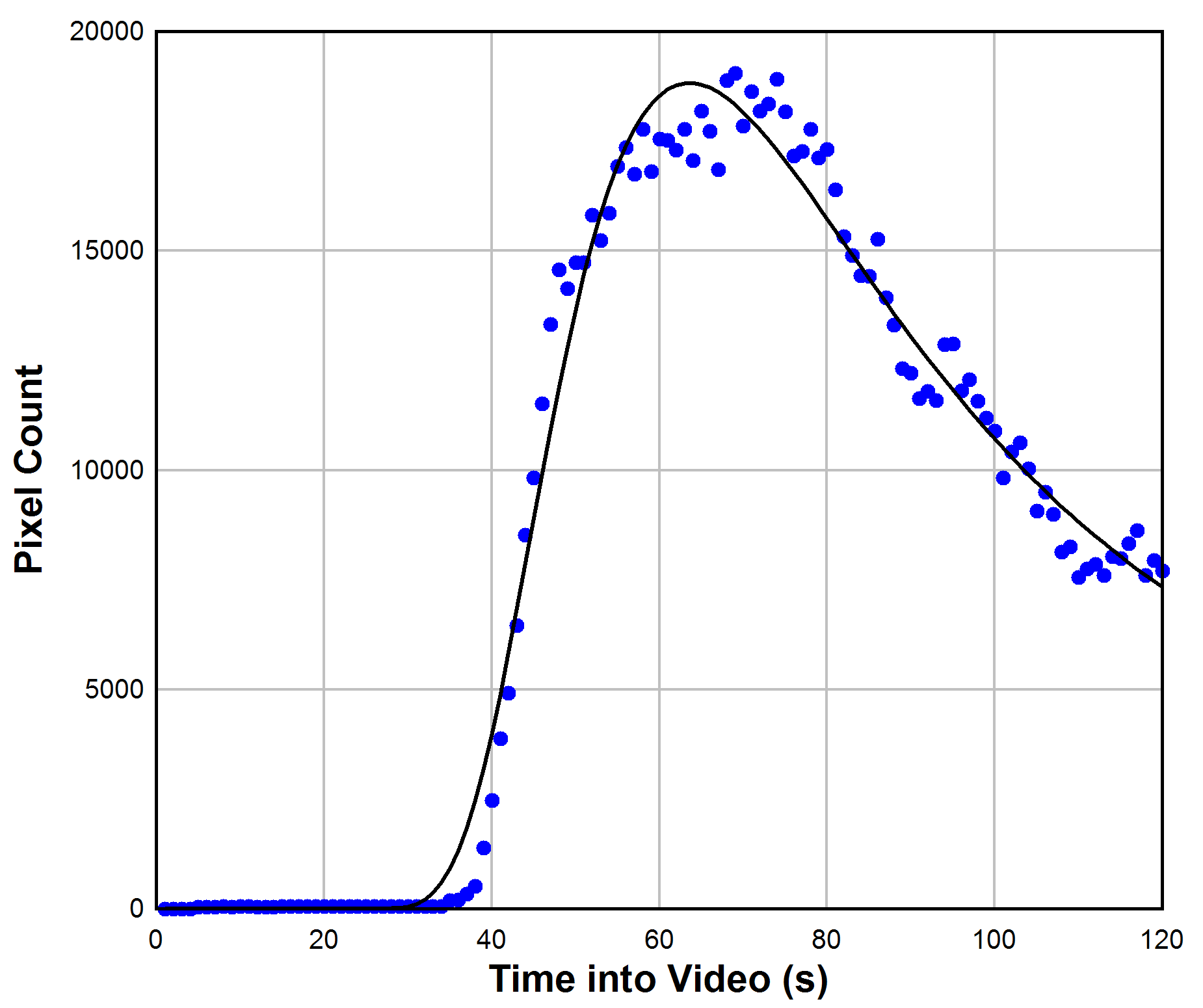


**Figure S5.** The variation in the abundance of single worms passing through the water column measured as the number of pixels in a frame occupied by worms; the time represents the 120 s duration of the run featuring floc #22; blue points: data; black line: associated fit based on a Gaussian speed distribution. Worms in the 3-D water column could overlap on the 2-D image and pixel count is not directly convertible to worm count.


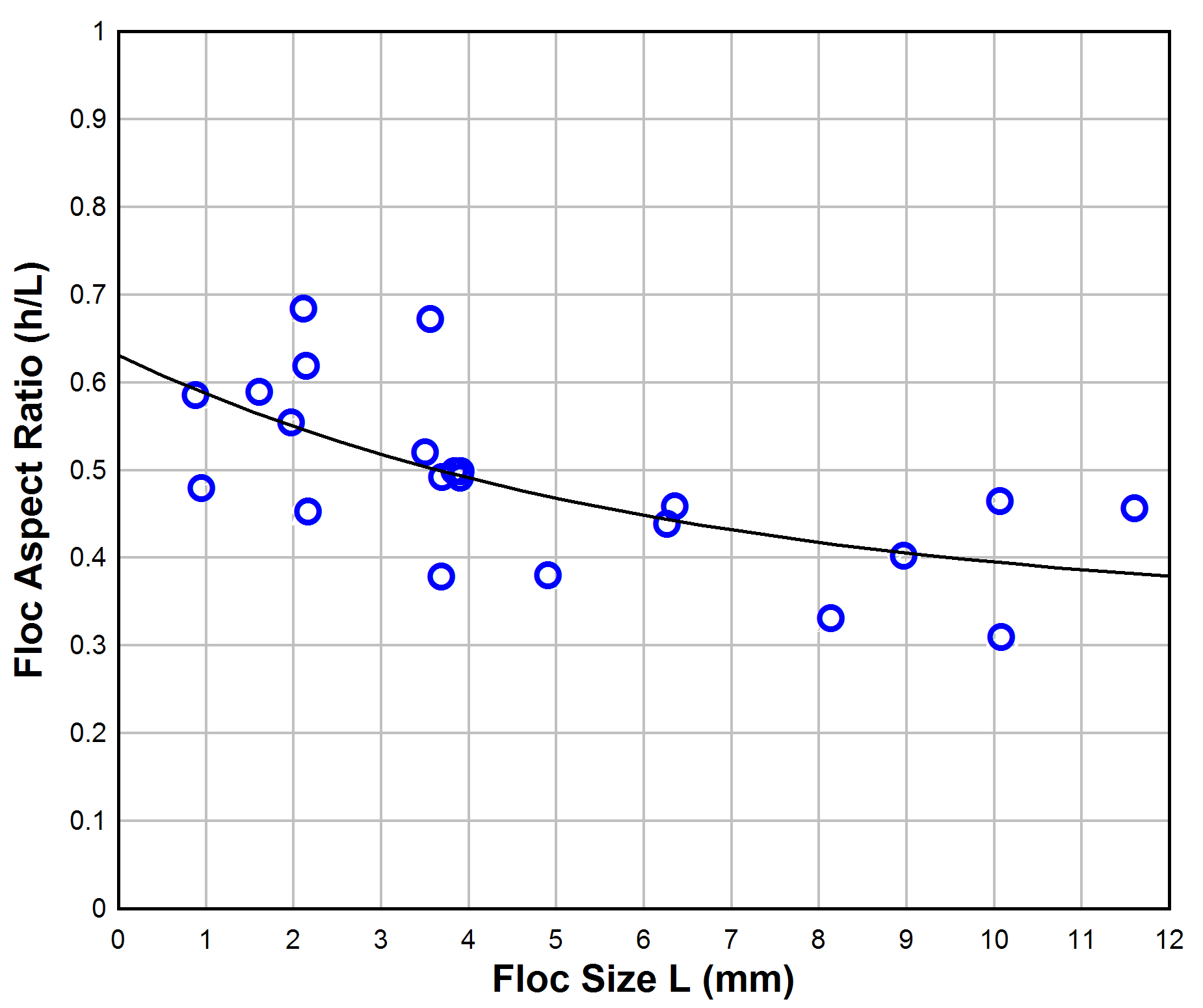


**Figure S6.** Aspect ratio h/L vs. L for the 22 flocs, where h and L are the mean minor and major axes, respectively, of the fitted ellipse. The fitted line is: h/L = 0.336+ 0.295exp(-0.160L).

**Section S4. Holes in the centres of toroidal flocs**


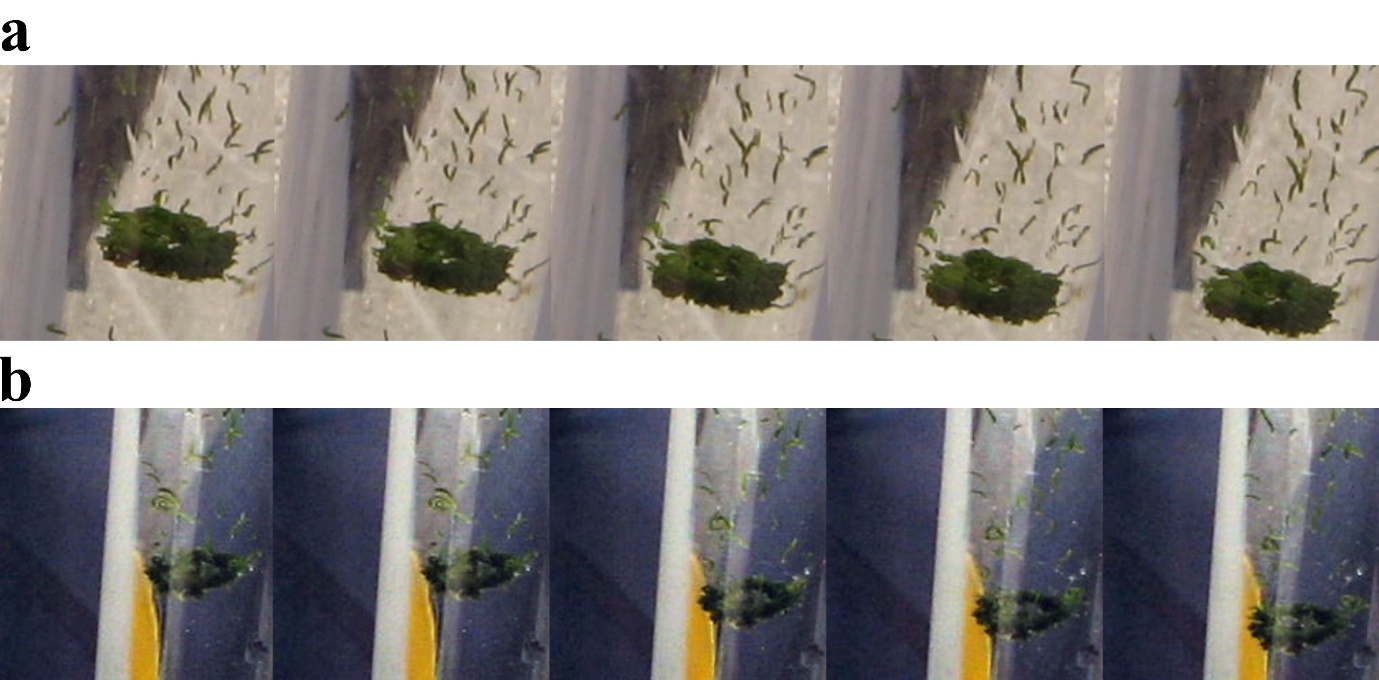


**Figure S7.** Oblique views of toroidal flocs. Both sequences *(a)* and *(b)* were taken with a hand-held Canon G7 camera. These photographs show the holes in the centres of toroidal flocs.

**References**

1. Franks NR *et al.* 2016 Social behaviour and collective motion in plant-animal worms. *Proc. R. Soc. B Biol. Sci.* **283**, 20152946. (doi:10.1098/rspb.2015.2946)
